# Efficacy and safety of fecal microbiota transplantation by washed preparation in patients with moderate to severely active ulcerative colitis

**DOI:** 10.1111/1751-2980.12938

**Published:** 2020-09-29

**Authors:** Min Chen, Xiao Lei Liu, Yu Jie Zhang, Yong Zhan Nie, Kai Chun Wu, Yong Quan Shi

**Affiliations:** ^1^ State Key Laboratory of Cancer Biology, National Clinical Research Center for Digestive Diseases and Xijing Hospital of Digestive Diseases Fourth Military Medical University Xi'an Shaanxi Province China; ^2^ Department of Medical Insurance Xijing Hospital, Air Force Military Medical University Xi'an Shaanxi Province China

**Keywords:** efficacy, fecal microbiota transplantation, safety, severe, ulcerative colitis

## Abstract

**Objective:**

We aimed to evaluate the short‐term efficacy and safety of fecal microbiota transplantation (FMT) by washed preparation for moderate to severely active UC.

**Methods:**

An open‐label prospective trial was conducted in an inflammatory bowel disease (IBD) tertiary referral center from April 2016 to March 2018. Patients with moderate to severely active UC were randomly assigned to undergo FMT thrice on day 1, 3 and 5 by nasojejunal tube (NJT) or transendoscopic enteral tubing (TET). The primary end‐point was a clinical response at week 2 post‐FMT. The secondary end‐points were clinical and endoscopic remission at week 12 post‐FMT, safety and disease progression.

**Results:**

Of the nine patients included, 77.8% (7/9) achieved a clinical response at week 2. And 55.6% (5/9) and 33.3% (3/9), respectively, achieved clinical remission and endoscopic remission at week 12. In two patients who had no response to FMT, one switched to anti‐tumor necrosis factor‐α therapy, and the other underwent a colectomy. FMT was delivered through NJT in 44.4% (4/9) of the patients, while TET was used in 55.6% (5/9). The clinical outcomes did not differ significantly based on the delivery route (*P* > 0.05). Adverse events, all mild and self‐limiting, were observed in 33.3% (3/9) of the patients.

**Conclusions:**

FMT by washed preparation appears to be a safe and effective adjunct therapy for moderate to severely active UC during a short‐term follow‐up. The efficacy did not differ significantly between the NJT or TET delivery routes. Further randomized controlled studies are needed to confirm these findings.

## 1 INTRODUCTION

Fecal microbiota transplantation (FMT) is very efficacious for the treatment of recurrent *Clostridium difficile* (*C. difficile*) infection, with the ability to restore a healthy microbial ecology and a mean cure rate of 87%‐90%.^1^, ^2^ In 2013, FMT has been suggested by the guidelines in the United States for the clinical treatment of *C. difficile* infection.^3^ However, the efficacy of FMT for treating ulcerative colitis (UC) remains controversial. The etiopathogenesis of UC is thought to be multifactorial, and alterations in the intestinal microbiome are one of its main characteristics. Moreover, there is more resistance to the reversal of enteric microbiota alterations in UC than in *C. difficile* infection.

In recent years, several randomized placebo‐controlled trials (RCTs) have evaluated the efficacy and safety of FMT for treating patients with mild to moderately active UC. In 2015 two clinical RCTs investigating the role of FMT in treating UC were published, but they reported conflicting outcomes. The first study^4^ including 75 patients administered weekly enemas for 6 weeks and reported clinical remission rates of 24% in the FMT group compared with 5% in the placebo control group at week 7. The second study^5^ including 50 patients which delivered FMT via a nasoduodenal tube at week 0 and week 3 found no significant difference in the achievement of clinical remission between participants who received fecal transplants from healthy donors and those who received autologous stool. Differences in patient populations, dosing regimens and delivery modalities may account for these conflicting results. In 2017 another RCT which administered FMT via a colonoscopy infusion once, followed by enemas 5 days per week for 8 weeks, the steroid‐free clinical and endoscopic remission or response rate was 27% in the FMT group compared with 8% in the placebo group (*P* = 0.021).^6^ In 2018 an RCT was published as an abstract using FMT delivered by colonoscopy followed by daily FMT capsules.^7^ It used rationally selected donors with high stool butyrate and found that histological inflammation decreased in 4/6 (66.7%) participants in the FMT arm compared with 1/6 (16.6%) in the placebo arm at week 12. In 2019, an RCT was published (N = 73) in which FMT was administered (using anaerobically prepared stool) via a colonoscopy followed by two enemas over 7 days. The steroid‐free remission rate was 32% in the group that received pooled donor FMT compared with 9% in the group that received autologous FMT (*P* = 0.03).^8^

Taken together, the evidence supports guarded optimism over FMT as a treatment option for UC. However, all these studies enrolled patients with mild to moderately active UC. There have been few studies designed to assess the efficacy and safety of FMT in patients with moderate to severely active UC. In addition, previous studies prepared fecal microbiota primarily by manual methods. However, this method has safety risks and presents challenges with regard to the psychological endurance and level of treatment acceptance of doctors, patients and donors. A washed preparation avoids these defects. Population‐based studies have shown that a washed preparation can significantly reduce FMT‐related adverse events.^9^ Therefore, we have prepared feces with a washed preparation using the automatic purification machine GenFMTer (FMT Medical, Nanjing, Jiangsu Province, China) since April 2016.

Therefore, we aimed to assess the short‐term efficacy and safety of FMT by a washed preparation in patients with moderate to severely active UC. All patients were randomly assigned to receive FMT via a nasojejunal tube (NJT) or transendoscopic enteral tubing (TET) and were followed up for 12 weeks.

## 2 PATIENTS AND METHODS

### 2.1 Study design

This was a single‐center, open‐label prospective study designed to determine the clinical efficacy and safety of FMT by washed preparation in the treatment of adult patients with moderate to severely active UC. The study was conducted at the Xijing Hospital of Digestive Diseases affiliated to the Air Force Military Medical University (Xi'an, Shaanxi Province, China), an IBD tertiary referral center in northwest China. The study was approved by the Local Ethics Committee of Xijing Hospital (LL‐KY‐20150305) and was registered in the ClinicalTrials.gov database (NCT04294615). All patients provided their written, informed consent before participating in the study.

### 2.2 Patient selection

Inpatients at the Xijing Hospital of Digestive Diseases were prospectively recruited from April 2016 to March 2018. Patients’ inclusion criteria included the following: (a) all patients were 18–74 years old at the time of enrollment; (b) the diagnosis of UC was made by a primary gastroenterologist based on the patient's medical history, physical examination, laboratory test results, radiological findings and gastrointestinal histologic evidence according to the Second European evidence‐based consensus;^10^ (c) moderate to severely acitve UC, defined as a Mayo score between 6 and 12 and an endoscopic subscore ≥2; and (d) for patients on medications, a stable dose had to be maintained for 1 week before entry into the study. The exclusion criteria included: (a) patients with a current or past intra‐abdominal abscess, acute abdomen or other clinical emergency requiring emergency management; (b) those who were pregnant; (c) with a prior history of FMT; (d) a prior history of tumor necrosis factor (TNF) inhibitor therapy; or (e) with other serious systemic diseases. The patients were allowed to continue to use other IBD medications, including corticosteroids and immunomodulators, during the study. However, we did not allow rectal therapies, including corticosteroids or 5‐aminosalicylate during the study.

The following laboratory examinations were offered to the eligible patients: (a) a blood biochemical examination, such as a complete blood count, albumin, erythrocyte sedimentation rates (ESR), C‐reactive protein (CRP); (b) screening for infectious diseases, such as screening for hepatitis A, B and C, human immunodeficiency virus (HIV); (c) screening for viruses, bacteria and parasites, such as Epstein‐Barr virus, cytomegalovirus, stool bacterial culture; and (d) stool tests for *C. difficile* infection, ova and parasites.

### 2.3 Donor selection

The study donors were all healthy, unrelated adults. Donors were regarded as standard donors when their fecal microbiota analysis was relatively stable over seven consecutive tests. Each patient was assigned to a single FMT donor.

The donors were carefully screened, and the exclusion criteria were as follows: (a) with a history of drug use (eg, antibiotics, laxatives or diet pills within the previous 3 months, or the use of immunomodulator or chemotherapy in the past); (b) with a history of disease, including but not limited to malignant neoplasms, infectious diseases, metabolic diseases (obesity, diabetes, metabolic syndrome), immune‐related diseases (inflammatory bowel disease, autoimmune disease, immunocompromised status, allergy), functional diseases (constipation, irritable bowel syndrome, chronic diarrhea) or other diseases such as colorectal polyps or those related to the disturbance of the intestinal microbiota; or (c) with a history of gastrointestinal operation. All donors underwent laboratory tests, such as regular blood parameters, CRP, ESR, immunoglobulin subtypes, biochemical parameters, hepatitis‐associated indices, HIV, syphilis, cytomegalovirus, Epstein‐Barr virus, rubella virus, herpes simplex virus, toxoplasma, and stool parameters (including stool bacterial culture, ova and parasites).

### 2.4 Fecal microbiota transplantation

We have been preparing the fecal microbiota with a washed preparation since April 2016. This process, recently named washed microbiota transplantation,^11^ was originally designed using an automatic microfiltration machine (GenFMTer) and subsequent repeated centrifugation plus suspension, with support from specific facilities. This washing preparation makes it possible to deliver a precise dose of the enriched microbiota instead of relying on the weight of stool. We followed the 1‐h FMT protocol, which means that the time from the collection of the stool from the donor to the transplantation of the microbiota into the patient's intestine is less than 1 hour.

All patients meeting the enrollment criteria underwent a standard polyethylene glycol‐based bowel preparation on the day prior to the FMT. No antibiotic pretreatment was administered before the FMT. There were two different delivery routes used for transplantation in this study, and the route was selected randomly by using a computer‐generated random number table. A NJT was fixed to the jejunum under gastroscopic guidance, and the location was confirmed by X‐ray examination. TET was fixed to the cecum with clips under colonoscopic guidance. The purified fecal microbiota was delivered to the intestine via NJT or TET. Approximately 200‐250 mL of fresh fecal suspension was injected over the course of more than 1 minute to avoid abdominal discomfort. The patient was required to remain in the right lateral position for 30 minutes after the FMT. Each patient received FMT thrice on days 1, 3 and 5.

### 2.5 Outcome measures

Patients were followed up until 12 weeks after the final FMT. The primary end‐point was the clinical response at 2 weeks after the FMT, defined as a reduction in the Mayo score of ≥3 and ≥30% from baseline, with a decrease in the rectal bleeding subscore of ≥1 or a subscore ≤1. The secondary end‐points were clinical remission (Mayo score ≤2, with no subscore >1) at week 12 post‐FMT, endoscopic remission (Mayo score = 0) at week 12 post‐FMT, safety and disease progression (measured by the initiation of anti‐TNF‐α treatment or switching to a colectomy). In this study, safety was evaluated by assessing the adverse events that occurred within 24 hours of the FMT and then weekly until week 12 post‐FMT. The severity of adverse events and the degree to which the event was related to the treatment were graded using U.S. National Cancer Institute criteria.^12^

### 2.6 Statistical analysis

Statistical analysis was performed using SPSS software 26.0 (IBM, Armonk, NY, USA). Descriptive statistics are described as the mean ± standard deviation or median and range, whereas categorical variables are expressed as numbers and percentages. Differences in baseline descriptive variables were assessed using the independent *t*‐test. We assessed categorical data using the χ^2^ test and Fisher's exact test. A two‐sided *P* value of less than 0.05 was regarded as statistically significant.

## 3 RESULTS

### 3.1 Study enrollment and patients’ characteristics

A total of nine patients were included in the study whose median age at the time of FMT was 47.90 ± 10.6 years (range 31‐61 y). They received FMT from two standard donors. Seven patients were men, and the other two were women. Their median disease duration of UC prior to the FMT was 5 years (range 0.2‐16 y). According to the Montreal classification for defining the disease extent, eight patients were classified as E3 (extensive colon) and the other was classified as E2 (left‐sided colon). With regard to the severity of the disease according to the Mayo score, six patients were at the severe disease stage and three were at the moderate stage. At the time of the FMT, six patients were receiving oral mesalamine, two patients were receiving intravenous steroids and one with steroid‐dependent UC was receiving oral mesalamine plus oral steroids. With regard to the FMT delivery route, five patients received the FMT via TET, and four patients received FMT via NJT. The baseline clinical characteristics of the participants are presented in Table 1.

**TABLE 1:** Baseline characteristics of patients prior to fecal microbiota transplantation

Patient	Sex	Age (y)	Disease duration (y)	Montreal classification	Disease severity	Concomitant medications	Delivery route
1	M	44	6	E3	Severe	Mesalamine (oral)	TET
2	F	56	6	E3	Severe	Steroids (intravenous)	NJT
3	M	58	16	E3	Moderate	Mesalamine (oral) + steroids (oral)	TET
4	M	52	5	E3	Severe	Mesalamine (oral)	TET
5	M	41	10	E2	Severe	Mesalamine (oral)	NJT
6	M	31	0.9	E3	Moderate	Mesalamine (oral)	TET
7	M	53	0.2	E3	Severe	Steroids (intravenous)	NJT
8	M	35	3	E3	Severe	Mesalamine (oral)	TET
9	F	61	1	E3	Moderate	Mesalamine (oral)	NJT

Abbreviations: E2, left‐sided colon; E3, extensive colon; F, female; M, male; NJT, nasojejunal tube; TET, transendoscopic enteral tubing.

### 3.2 Clinical outcomes of FMT

**FIGURE 1:**
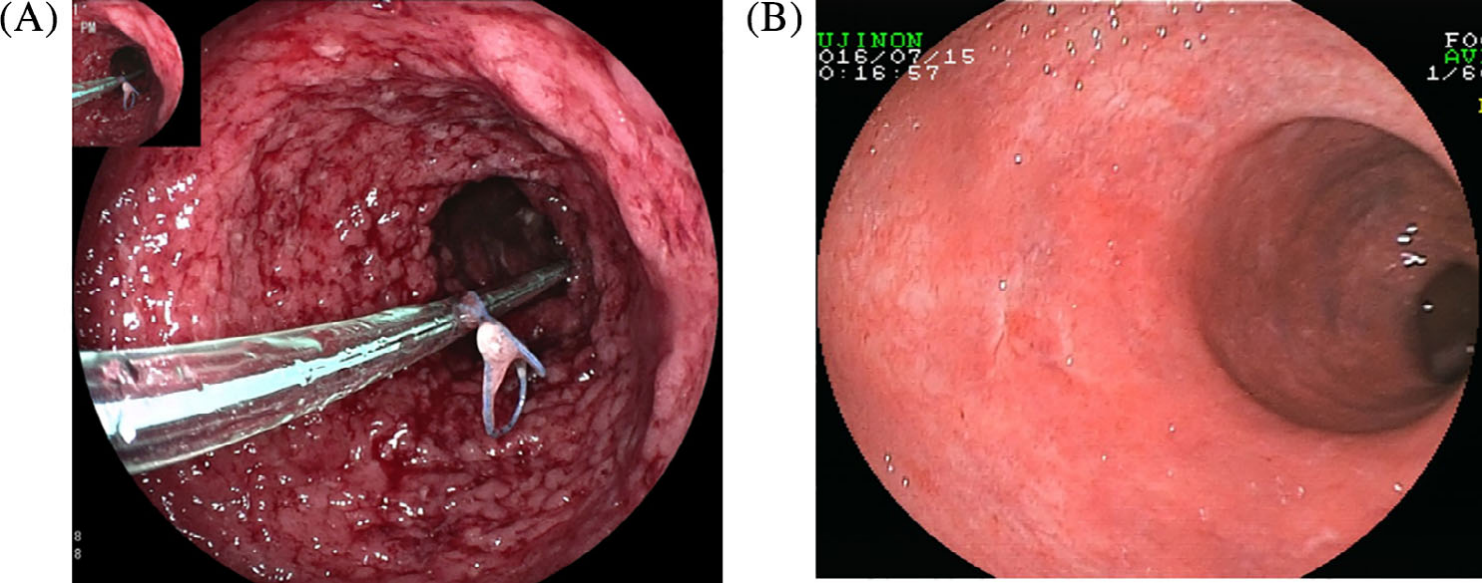
Endoscopic images before and after fecal microbiota transplantation (FMT) via transendoscopic enteral tubing. A, Transendoscopic enteral tubing fixed to the colon by clips before FMT. B, Endoscopic remission at week 12 after FMT [Color figure can be viewed at wileyonlinelibrary.com]

**FIGURE 2:**
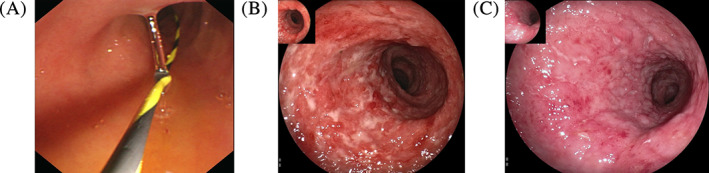
Endoscopic images before and after fecal microbiota transplantation (FMT) via nasojejunal tube. A, Nasojejunal tube fixed to jejunum before FMT. B, Severe ulcerative colitis before FMT. C, Endoscopic improvement at week 12 after FMT [Color figure can be viewed at wileyonlinelibrary.com]

At two weeks post‐FMT, a clinical response was reported in seven (77.8%) of the nine patients based on the Mayo score. And at week 12 post‐FMT, clinical remission was achieved in five (55.6%) of the nine patients, whereas endoscopic remission was achieved in three (33.3%). However, two patients (patient numbers 5 and 7) did not respond at both week 2 and week 12. Notably, the patient with steroid‐dependent UC achieved steroid‐free clinical remission and endoscopic remission (patient number 3). Two patients maintained long‐term clinical and endoscopic remission until 6 months after FMT (patient numbers 4 and 6). One patient was switched to anti‐TNF‐α therapy (patient number 5), and the other underwent a colectomy (patient number 7). Representative endoscopic images are shown in Figures 1 and 2.

In addition, laboratory indexes, including the levels of CRP, ESR, hemoglobin, albumin and platelet count, were also evaluated. All but two patients had improvements in or normalization of their CRP levels at the week 2 post‐FMT. Their median CRP level decreased from 18.3 mg/L at baseline to 9.39 mg/L at week 2 post‐FMT and then to 1.31 mg/L at week 12 post‐FMT. With regard to other indexes, the mean ESR decreased from 31.7 ± 12.8 mm/h at baseline to 18.7 ± 15.1 mm/h at week 2 post‐FMT and then to 7.0 ± 6.5 mm/h at week 12 post‐FMT. The mean hemoglobin level increased from 109.1 ± 23.7 g/L at baseline to 131.1 ± 24.2 g/L at week 12 post‐FMT. The mean platelet count decreased from (302.3 ± 73.5) × 10^9^/L at baseline to (282.7 ± 90.4) × 10^9^/L at week 2 post‐FMT and then to (213.1 ± 42.0) × 10^9^/L at week 12 post‐FMT. While the mean albumin level increased from 30.8 ± 6.9 g/L at baseline to 37.1 ± 4.7 g/L at week 2 post‐FMT and then to 41.5 ± 5.8 g/L at week 12 post‐FMT. Patients’ clinical outcomes are summarized in Tables 2 and 3.

**TABLE 2:** Clinical and laboratory indexes before and after fecal microbiota transplantation

Patient no.	Follow‐up	Total Mayo score	CRP (mg/L)	ESR (mm/h)	Hemoglobin (g/L)	Platelet (×10^9^/L)	Albumin (g/L)	Clinical response at wk 2	Clinical remission at wk 12	Endoscopic remission at wk 12	Switched to anti‐TNF‐α/colectomy
1	Baseline	11	39.1	31	107	473	30.7	Yes	Yes	No	No
	Wk 2	7	9.7	14	100	436	33.5				
	Wk 12	2	3.2	5	135	256	38.0				
2	Baseline	12	53.2	51	70	296	18.3	Yes	No	No	No
	Wk 2	7	16.4	3	82	131	38.3				
	Wk 12	4	5.2	4	90	150	37.5				
3	Baseline	8	12.4	33	130	277	39.1	Yes	Yes	Yes	No
	Wk 2	5	0.658	12	125	245	39.6				
	Wk 12	2	0.5	7	133	238	39.4				
4	Baseline	11	18.3	25	112	297	31.5	Yes	Yes	Yes	No
	Wk 2	7	9.39	17	148	327	36.0				
	Wk 12	1	0.192	1	160	215	45.5				
5	Baseline	11	4.65	32	97	290	30.5	No	No	No	Anti‐TNF‐α therapy
	Wk 2	10	5.7	30	85	295	32.9				
	Wk 12	NA	NA	NA	NA	NA	NA				
6	Baseline	7	0.785	5	138	253	40.2	Yes	Yes	Yes	No
	Wk 2	2	0.157	2	141	202	45.4				
	Wk 12	0	0.232	1	153	190	53.0				
7	Baseline	12	33.9	43	78	322	24.8	No	No	No	Colectomy
	Wk 2	12	47	50	65	344	30.2				
	Wk 12	NA	NA	NA	NA	NA	NA				
8	Baseline	11	20.7	28	130	201	34.8	Yes	No	No	No
	Wk 2	7	2.75	12	128	232	36.5				
	Wk 12	4	4.95	12	137	180	38.4				
9	Baseline	9	5.66	37	120	312	27.2	Yes	Yes	No	No
	Wk 2	6	13.4	28	114	332	41.7				
	Wk 12	2	1.31	19	110	263	38.5				

Abbreviations: CRP, C‐reactive protein; ESR, erythrocyte sedimentation rates; NA, TNF, tumor necrosis factor.

**TABLE 3:** Clinical outcomes in recipients of fecal microbiota transplantation

Outcome	n/N (%)
Clinical response at wk 2	7/9 (77.8)
Clinical remission at wk 12	5/9 (55.6)
Endoscopic remission at wk 12	3/9 (33.3)
Switched to anti‐TNF‐α therapy	1/9 (11.1)
Colectomy	1/9 (11.1)

Abbreviations: TNF, tumor necrosis factor.

We then compared the clinical outcomes between patients who received the FMT via different delivery routes, that is, TET or NJT. There were no significant differences between the two groups in baseline characteristics including their age, disease duration and Mayo score before FMT. Regarding the primary end‐point, all five patients (100%) in the TET group and two of the four patients (50%) in the NJT group achieved a clinical response at week 2 post‐FMT. With regard to the secondary end‐points, four (80.0%) of the five patients in the TET group and one (25.0%) of the four patients in the NJT group were in clinical remission at week 12 post‐FMT. Three (60.0%) of the five patients in the TET group and none in the NJT group was in endoscopic remission at week 12 post‐FMT. Compared with the NJT group, although the TET group had a higher clinical response rate at week 2 and higher clinical and endoscopic remission rates at week 12, there were no significant differences between the two groups in any of the clinical outcomes (*P* > 0.05). The results are summarized in Table 4.

**TABLE 4:** Clinical outcomes stratified by fecal microbiota transplantation (FMT) delivery routes

Delivery route	Age, y (mean ± SD)	Disease duration, y (median [range])	Mayo score before FMT (mean ± SD)	Clinical response at wk 2, n/N (%)	Clinical remission at wk 12, n/N (%)	Endoscopic remission at wk 12, n/N (%)
TET (n = 5)	44.0 ± 11.3	5 (0.9‐16)	9.6 ± 1.9	5/5 (100)	4/5 (80.0)	3/5 (60.0)
NJT (n = 4)	52.8 ± 8.5	3.5 (0.2‐10)	11.0 ± 1.4	2/4 (50.0)	1/4 (25.0)	0/4 (0)
*P* value	0.443	0.919	0.171	0.167	0.206	0.167

Abbreviations: NJT, nasojejunal tube; SD, standard deviation; TET, transendoscopic enteral tubing.

### 3.3 Adverse events

No serious adverse events directly due to FMT occurred in any of the patients. Mild adverse events related to FMT were seen in three patients (33.3%; patient numbers 2, 5 and 9), including mild abdominal pain, diarrhea and fatigue, all of which were self‐limiting. All three patients with mild adverse events received FMT therapy via NJT. The symptoms occurred on the day of FMT and disappeared spontaneously the next day. The other patients had no complaints after FMT. And none of the patients had any infectious complications due to FMT.

## 4 DISCUSSION

To our knowledge, although one retrospective case series has reported patients with moderate to severe medically refractory UC who were treated with fecal bacteriotherapy in 2003,^13^ our study is the first open‐label prospective study to evaluate the efficacy and safety of FMT as an adjunct therapy for patients with moderate to severely active UC and compared the efficacy of two different delivery routes of FMT. In this study we prepared the fecal microbiota using a washed preparation method and administered the FMT via a NJT or TET, and the procedure was safe and well tolerated.

The short‐term clinical response is very important to evaluate the treatment efficacy for patients with moderate to severely active UC because these patients need to switch to another therapy as soon as possible if the current therapeutic regimen is not effective. Therefore, the primary end‐point of our study was the clinical response at week 2 post‐FMT. We found that FMT was associated with a clinical response at week 2 post‐FMT in 77.8% (7/9) of the patients. In particular, 66.7% (4/6) of the patients with severe UC achieved a clinical response at week 2, and 3 out of these four patients did not use steroids. With regard to the two (22.2%) non‐responders, one switched to anti‐TNF‐α therapy, and the other underwent a colectomy. This result seems better than that previously reported in a systematic review of 32 trials of steroid therapy for acute severe UC, with an overall response to intravenous steroids of 67% (95% confidence interval [CI] 65‐69%).^14^ This shows that FMT may be effective as an adjunct therapy to induce rapid remission in patients with acute moderate to severe UC, especially those with severe UC. At week 12 post‐FMT, 55.6% (5/9) and 33.3% (3/9) of our patients had achieved clinical and endoscopic remission, respectively. These rates were 33.3% (2/6) and 16.7% (1/6), respectively, in patients with severe UC. In particular, one patient with steroid‐dependent UC with a disease duration of 16 years achieved steroid‐free clinical and endoscopic remission. This result is similar to those in previous studies, which also found that FMT may be a promising therapeutic option for patients with steroid‐dependent UC.^15^, ^16^ Meanwhile, we also found that two (66.7%) of the three patients who achieved endoscopic remission maintained long‐term remission until 6 months after FMT, and one of them had severe UC.

To date, an increasing number of studies have focused on the efficacy of FMT as a treatment for UC. However, the response rates of the patients vary significantly from 0% to 67%.^4^, ^17^, ^18^, ^19^ There are a number of possible reasons for such variability, such as sample size, selection criteria, delivery route, dose and frequency of FMT, and the evaluation index. Among them, methodological differences, including the delivery route of FMT (via the upper or lower gastrointestinal tract), may be related to the discrepancies in the results. In our study we compared clinical outcomes between two different FMT delivery routes, that is, by NJT or TET. Although much higher clinical and endoscopic response or remission rates were seen in the TET group, there were no significant differences in primary and secondary end‐points between the two groups. This result indicates that the delivery route may have no obvious effect on the use of FMT to treat UC. Further studies with large sample sizes are needed to confirm this hypothesis. Interestingly, we found some differences between the two delivery routes. First, mild adverse events, which were self‐limiting, were only observed in patients received FMT via NJT. Second, patients who received FMT via TET felt more comfortable because it did not affect their eating and breathing. However, the NJT could be maintained for a longer time than TET if the patient could tolerate it. All this indicates that the delivery route should be considered comprehensively in the clinical setting.

Furthermore, we prepared the fecal microbiota with a washed preparation using an automatic microfiltration machine. This method avoids the discomfort experienced by doctors and patients during the process of preparing and infusing the fecal microbiota and simultaneously decreased the incidence of adverse events due to FMT. In a previous study it was shown that the rate of adverse events in patients undergoing FMT prepared manually was 21.7%, which was significantly higher than the 8.7% in those who underwent FMT prepared with a washed preparation.^20^ In our study, FMT by washed preparation was well tolerated. The overall incidence of adverse events was 33.3% (3/9), and all adverse events were mild and self‐limiting, including abdominal pain, diarrhea and fatigue. Although serious events including aspiration, infection and IBD flares have been previously reported,^2^, ^21^, ^22^ we did not observe any serious adverse events or infectious complications directly related to FMT.

Our study had some limitations. First, the sample size was too small to draw a solid conclusion about the effects of FMT on patients with moderate to severely active UC. Second, because it was relatively difficult to obtain written informed consent from severely active patients with UC if there was a possibility of being assigned to the placebo group, a placebo control group was not included in our study. Finally, the levels of noninvasive biomarkers such as calprotectin were not determined in all patients.

In conclusion, our study used a washed preparation to prepare the fecal microbiota and analyzed the efficacy and safety of FMT for the treatment of moderate to severely active UC, showing that there was a high short‐term response and remission rates after FMT. Notably, FMT may be an effective therapy for the steroid‐dependent UC group. Additionally, we compared the clinical outcomes between different delivery routes, NJT and TET, and found no significant differences. All the patients tolerated the treatment well and had no serious adverse events. These data suggest that FMT as an adjunct therapy is safe and effective for patients with moderate to severely active UC. Further randomized controlled trials with large sample sizes are needed to confirm our findings, and longer follow‐up will be needed to assess its long‐term efficacy and safety.

## CONFLICTS OF INTEREST

The authors have no conflicts of interest to declare.
